# 1-year risks of cancers associated with COVID-19 vaccination: a large population-based cohort study in South Korea

**DOI:** 10.1186/s40364-025-00831-w

**Published:** 2025-09-26

**Authors:** Hong Jin Kim, Min-Ho Kim, Myeong Geun Choi, Eun Mi Chun

**Affiliations:** 1 Department of Orthopedic Surgery, Kyung-in Regional Military Manpower Administration, Suwon, Korea; 2 Informatization Department, Ewha Womans University Seoul Hospital, Seoul, Republic of Korea; 3 Division of Pulmonary and Critical Care Medicine, Department of Internal Medicine, Ewha Womans University Mokdong Hospital, 1071, Anyangcheon-ro, Yangcheon-gu, Seoul, 07985 Republic of Korea

**Keywords:** Cancer, COVID-19, Vaccine, mRNA-based vaccine, cDNA-based vaccine

## Abstract

The oncogenic potential of SARS-CoV-2 has been hypothetically proposed, but real-world data on COVID-19 infection and vaccination are insufficient. Therefore, this large-scale population-based retrospective study in Seoul, South Korea, aimed to estimate the cumulative incidences and subsequent risks of overall cancers 1 year after COVID-19 vaccination. Data from 8,407,849 individuals between 2021 and 2023 were obtained from the Korean National Health Insurance database. The participants were categorized into two groups based on their COVID-19 vaccination status. The risks for overall cancer were assessed using multivariable Cox proportional hazards models, and data were expressed as hazard ratios (HRs) and 95% confidence intervals (CIs). The HRs of thyroid (HR, 1.351; 95% CI, 1.206–1.514), gastric (HR, 1.335; 95% CI, 1.130–1.576), colorectal (HR, 1.283; 95% CI, 1.122–1.468), lung (HR, 1.533; 95% CI, 1.254–1.874), breast (HR, 1.197; 95% CI, 1.069–1.340), and prostate (HR, 1.687; 95% CI, 1.348–2.111) cancers significantly increased at 1 year post-vaccination. In terms of vaccine type, cDNA vaccines were associated with the increased risks of thyroid, gastric, colorectal, lung, and prostate cancers; mRNA vaccines were linked to the increased risks of thyroid, colorectal, lung, and breast cancers; and heterologous vaccination was related to the increased risks of thyroid and breast cancers. Given the observed associations between COVID-19 vaccination and cancer incidence by age, sex, and vaccine type, further research is needed to determine whether specific vaccination strategies may be optimal for populations in need of COVID-19 vaccination.

**Supplementary Information:**

The online version contains supplementary material available at 10.1186/s40364-025-00831-w.

To the editor

Since the Coronavirus disease 2019 (COVID-19) outbreak in December 2019, it has become a global concern because of the lack of preventive and treatment options. It is caused by severe acute respiratory syndrome coronavirus 2 (SARS-CoV-2), which is linked to high morbidity and mortality among the elderly [1, 2]. With the rapid development of COVID-19 vaccines, the fatal complications caused by COVID-19 have been alleviated; however, several other issues, including adverse events related to vaccines have emerged [3–6].

Similar to other viruses, such as human papillomavirus and Epstein–Barr virus, SARS-CoV-2 shows an oncogenic potential, which has been hypothetically proposed based on its mechanisms of action, including the renin–angiotensin–aldosterone system, viral mutagenicity, and inflammatory cascade [7]. Given the shared structures, such as the spike protein in COVID-19 vaccines, we further hypothesized that COVID-19 vaccines might potentially be associated with cancer risks; however, real-world data are insufficient [8]. In this population-based retrospective study, we estimated the cumulative incidences and risks of cancers 1 year after COVID-19 vaccination. In the South Korean cohort of 8,407,849 individuals between 2021 and 2023, we finally included 595,007 and 2,380,028 individuals after the 1:4 propensity score matching (PSM). For the vaccinated population, 355,896 and 711,792 individuals were included in the non-booster and booster groups, after the 1:2 PSM. The measured outcomes were cumulative incidences and corresponding risks of cancers at one year after COVID-19 vaccination, which was also stratified by the types of vaccine, sex and age (Additional File 1).

Our data showed associations between COVID-19 vaccination and an increased the risk of six cancer types, namely, thyroid (hazard ratio [HR], 1.35; 95% confidence interval [CI], 1.21–1.51), gastric (HR, 1.34; 95% CI, 1.13–1.58), colorectal (HR, 1.28; 95% CI, 1.12–1.47), lung (HR, 1.533; 95% CI, 1.25–1.87), breast (HR, 1.20; 95% CI, 1.07–1.34), and prostate (HR, 1.69; 95% CI, 1.35–2.11) cancers (Fig. 1 and Additional File 2). In terms of vaccine type, cDNA vaccines were associated with the increased risks of thyroid, gastric, colorectal, lung, and prostate cancers; mRNA vaccines were linked to the increased risks of thyroid, colorectal, lung, and breast cancers; and heterologous vaccination was related to the increased risks of thyroid and breast cancers. Meanwhile, vaccinated males were more vulnerable to gastric and lung cancers, whereas vaccinated females were more susceptible to thyroid and colorectal cancers. In terms of age stratification, the relatively younger population (individuals under 65 years) was more vulnerable to thyroid and breast cancers; by comparison, the older population (75 years and older) was more susceptible to prostate cancer (Additional File 3). Booster doses substantially affected the risk of three cancer types in the vaccinated population: gastric and pancreatic cancers (Table 1). Our findings highlighted various cancer risks associated with different COVID-19 vaccine types.

**Fig. 1:**
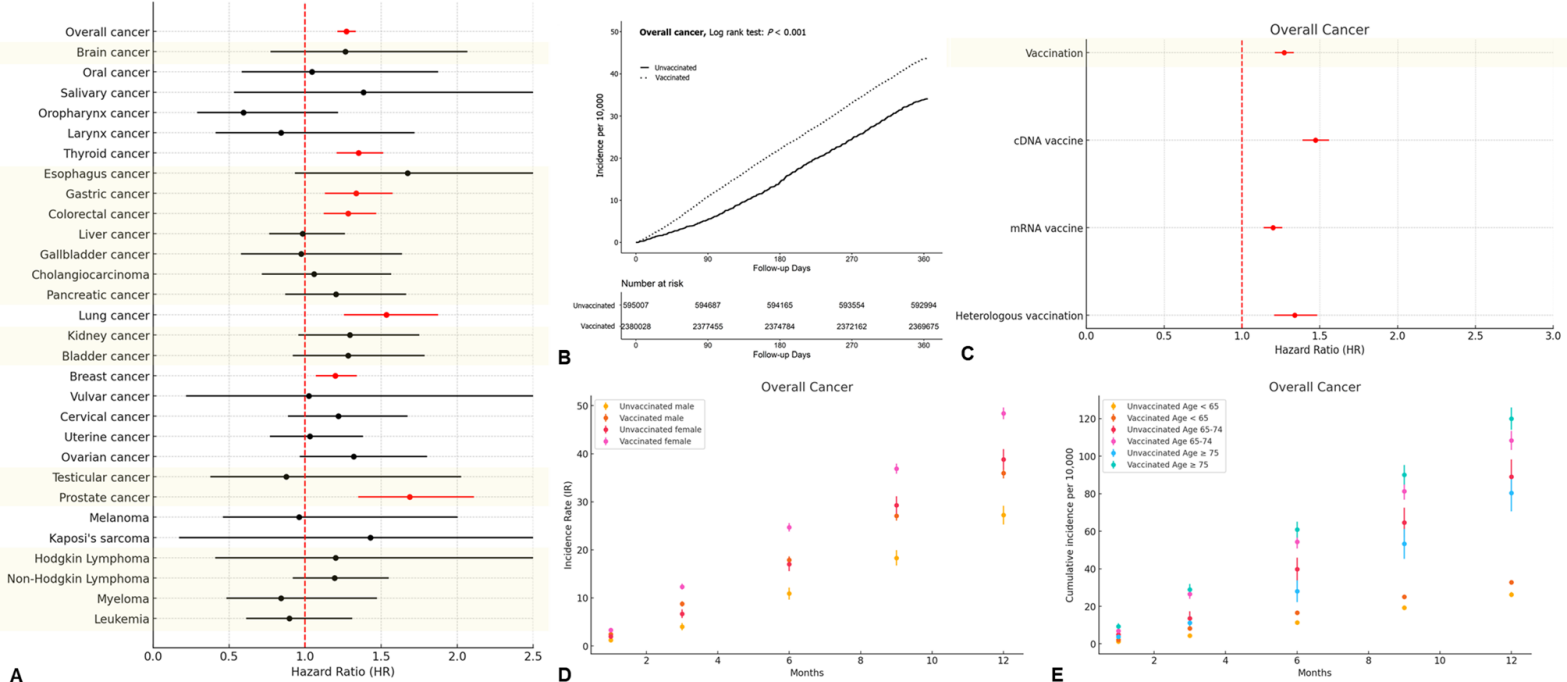
Risks of cancers associated with COVID-19 vaccines. (**A**) Hazard ratio of overall and 29 different types of cancers; (**B**) Cumulative incidences of overall cancers; (**C**) Hazard ratio of overall cancers according to the vaccine types; (**D**) Cumulative incidences of overall cancers stratified by sex; (**E**) Cumulative incidences of overall cancers stratified by age

**Table 1:** Risk of cancers for the matched vaccinated cohort according to the booster doses of COVID-19 vaccine

Cancer	HR (95% CI)	*P*-value	Cancer	HR (95% CI)	*P*-value
Overall cancer	1.01 (0.95–1.06)	0.803	Kidney cancer	0.92 (0.67–1.28)	0.618
Brain cancer	1.24 (0.68–2.27)	0.478	Bladder cancer	1.21 (0.81–1.82)	0.352
Oral cancer	0.94 (0.52–1.69)	0.839	Breast cancer	1.01 (0.89–1.13)	0.932
Salivary cancer	0.67 (0.28–1.59)	0.364	Vulvar cancer	0.34 (0.06–2.02)	0.233
Oropharynx cancer	0.80 (0.36–1.76)	0.578	Cervical cancer	1.27 (0.83–1.93)	0.274
Larynx cancer	0.93 (0.34–2.50)	0.877	Uterine cancer	0.86 (0.61–1.20)	0.360
Thyroid cancer	0.91 (0.79–1.03)	0.139	Ovarian cancer	0.88 (0.63–1.23)	0.449
Esophagus cancer	1.21 (0.58–2.54)	0.609	Testicular cancer	0.67 (0.15–3.01)	0.605
**Gastric cancer**	**1.23 (1.01–1.50)**	**0.041**	Prostate cancer	1.26 (0.95–1.66)	0.104
Colorectal cancer	1.00 (0.86–1.18)	0.962	Melanoma	1.00 (0.38–2.66)	0.997
Liver cancer	1.17 (0.85–1.62)	0.334	Kaposi’s sarcoma	N/A (no event)	N/A
Gallbladder cancer	0.68 (0.38–1.21)	0.186	Hodgkin lymphoma	3.01 (0.36–25.01)	0.308
Cholangiocarcinoma	1.55 (0.92–2.60)	0.097	Non-Hodgkin lymphoma	0.76 (0.57–1.00)	0.053
**Pancreatic cancer**	**2.25 (1.44–3.50)**	**< 0.001**	Myeloma	0.58 (0.31–1.07)	0.080
Lung cancer	0.96 (0.78–1.17)	0.677	**Leukemia**	**0.56 (0.35–0.92)**	**0.020**

Bold indicates the data for the statistical significance. HR, hazard ratio; CI, confidence interval

Given the limited availability of real-world data, our population-based cohort study in Seoul, South Korea suggested epidemiological associations between the cumulative incidence of cancers and COVID-19 vaccination, which varied by sex, age, and vaccine type. However, further studies are warranted to elucidate potential causal relationships, including the underlying molecular mechanisms related to COVID-19 vaccine–induced hyperinflammation.

The concept of a booster dose involves re-exposure to the immunizing antigen to enhance immunity [9]. The protective effect of COVID-19 vaccination diminishes over time; as such, further booster doses are needed to restore immunity [9, 10]. Given the decreasing severity of COVID-19, current concerns regarding the COVID-19 vaccine primarily revolve around AEs even with booster shots. Considering the significantly higher risk of gastric cancer in vaccinated individuals than in unvaccinated individuals, clinicians should prioritize monitoring the risk of gastric cancer in relation to COVID-19 booster doses.

In conclusion, COVID-19 vaccination could be associated with an increased risk of six specific cancer types, including thyroid, gastric, colorectal, lung, breast, and prostate cancers. Notably, this COVID-19 vaccination-associated cancer risk was likely more elevated among individuals aged ≤ 65 years except in individuals with prostate cancer. Given the observed associations between COVID-19 vaccination and cancer incidence by age, sex, and vaccine type, further research is needed to determine whether specific vaccination strategies may be optimal for populations in need of COVID-19 vaccination.

## Supplementary Information

Below is the link to the electronic supplementary material.

Supplementary Material 1

Supplementary Material 2

Supplementary Material 3

## Data Availability

The data that support the findings of this study are available from National Health Insurance Service in South Korea but restrictions apply to the availability of these data, which were used under license for the current study, and so are not publicly available. Data are however available from the authors upon reasonable request and with permission of National Health Insurance Service, South Korea.

## Abbreviations

COVID-19: Coronavirus disease 2019
SARS-CoV-2: Severe acute respiratory syndrome coronavirus 2
PSM: Propensity score matching
HR: Hazard ratio
CI: Confidence interval
