# Supplementary material for: 1-year risks of cancers associated with COVID-19 vaccination: a large population-based cohort study in South Korea
Source: Biomark Res. 2025 Sep 26;13:114. doi: 10.1186/s40364-025-00831-w (PMC12465339; doi:10.1186/s40364-025-00831-w)
Supplement: Supplementary file 1 — Supplementary Material 1 [file 40364_2025_831_MOESM1_ESM.docx]

**Additional File 2 : Detailed results of overall cancers for**

**“1-year risks of cancers associated with COVID-19 vaccination: A large population-based cohort study in South Korea “**

- List-

1. Detailed results of overall cancers for this study

2. Table S2: Baseline characteristics for main cohort after 1:4 propensity score matching

3. Table S3: Baseline characteristics for sub-cohort after 1:2 propensity score matching

4. Table S4: The data of cumulative incidences on overall cancers.

**1. Detailed Results**

**Baseline characteristics**

After 1:4 PSM, a total of 2,975,035 individuals with a mean age of 44.08 years (SD, 16.00) were included, and they comprised 1,385,745 males (46.58%) and 1,589,290 females (53.42%). In the South Korean vaccinated cohort (n = 2,380,028), the majority received the BNT162b2 vaccine as their first vaccination (1,603,518 individuals [67.37%]) and second vaccination (1,750,754 individuals [73.56%]). Regarding vaccine types between the first and second vaccinations, 1,928,363 individuals (81.02%) were treated with mRNA vaccines only, 333,698 individuals (14.02%) were given cDNA vaccines only, and 117,967 individuals (4.96%) were administered with heterologous vaccinations in the COVID-19 vaccinated cohort (**Table S2**). For the booster doses of 1:2 matched model, a total of 1,067,688 individuals with a mean age of 46.19 years (SD, 14.84) were included, and they comprised 425,055 males (39.81%) and 642,633 females (60.19%). The main portion of the booster vaccination was BNT162b2 (n = 623,688, 87.62%) in the booster group (**Table S3**).

**Relationship between overall cancer and COVID-19 vaccination**

The Cox proportional hazards regression analysis revealed that the 1-year HR for overall cancer incidence following COVID-19 vaccination was 1.27, with a 95% CI of 1.21–1.33. Our findings demonstrated that COVID-19 vaccination was significantly associated with an increased risk of cancer in six organs: the thyroid (HR, 1.35; 95% CI, 1.21–1.51), gastric (HR, 1.335; 95% CI, 1.13–1.58), colorectal (HR, 1.28; 95% CI, 1.12–1.47), lung (HR, 1.53; 95% CI, 1.25–1.87), breast (HR, 1.20; 95% CI, 1.07–1.34), and prostate (HR, 1.69; 95% CI, 1.35–2.11) (**Figure 1A**).

The cumulative incidence of overall cancers was significantly higher in the vaccinated group than in the unvaccinated group at 1 month (2.91 vs. 1.63), 3 months (10.66 vs. 5.45), 6 months (21.54 vs. 14.17), 9 months (32.33 vs. 24.18), and 1 year (42.62 vs. 33.43) post-vaccination (**Figure 1B**). For the vaccine type, the highest HR was observed in the cDNA vaccine-only group (1.472; 95% CI, 1.389–1.560), followed by heterologous vaccination (HR, 1.339; 95% CI, 1.208–1.484) and mRNA vaccine-only groups (HR, 1.199; 95% CI, 1.140–1.260; **Figure 1C**). When we stratified our data according to sex, the cumulative incidences of the overall cancers in males and females were significantly higher in the vaccinated group than in the unvaccinated group. The highest cumulative incidence (48.40; 95% CI, 47.20–49.61) was found in the vaccinated females 1 year following the COVID-19 vaccination (**Figure 1D**). It was also significantly higher in the vaccinated group than in the unvaccinated group regardless of age stratification. The highest cumulative incidence was detected in the vaccinated individuals aged ≥75 years (119.96; 95% CI, 114.01–125.92) 1 year following COVID-19 vaccination (**Figure 1E**). The data of cumulative incidences on overall cancers are illustrated in detail in **Table S4**. We specifically described the six COVID-19 vaccination-related cancers in the next sections.

**2. Table S2:** Baseline characteristics for main cohort after 1:4 propensity score matching

**Table S2** Baseline characteristics for the matched cohort stratified by COVID-19 vaccination

| **1:4 matched cohort** | **Total**  **(n = 2,975,035)** | **Unvaccinated group**  **(n = 595,007)** | **Vaccinated group**  **(n = 2,380,028)** | **SMD** |
| --- | --- | --- | --- | --- |
| Sex (n, %)  Male  Female | 1,385,745 (46.58%)  1,589,290 (53.42%) | 277,149 (46.58%)  317,858 (53.42%) | 1,108,596 (46.58%)  1,271,432 (53.42%) | < 0.001 |
| Age (mean ± SD, years)  < 65 years (n, %)  65 to 74 years (n, %)  ≥ 75 years (n, %) | 44.08 ± 16.00  2,613,610 (87.85%)  201,065 (6.76%)  160,360 (5.39%) | 44.08 ± 16.00  522,722 (87.85%)  40,213 (6.76%)  32,072 (5.39%) | 44.08 ± 16.00  2,090,888 (87.85%)  160,852 (6.76%)  128,288 (5.39%) | < 0.001  < 0.001 |
| Income (n, %)  Low  Middle  High | 761,071 (25.58%)  973,508 (32.72%)  1,240,456 (41.70%) | 176,983 (29.74%)  190,924 (32.09%)  227,100 (38.17%) | 584,088 (24.54%)  782,584 (32.88%)  1,013,356 (42.58%) | 0.090 |
| Charlson comorbidity index (n, %)  0  1  ≥ 2 | 2,562,915 (86.15%)  244,815 (8.23%)  167,305 (5.62%) | 512,583 (86.15%)  48,963 (8.23%)  33,461 (5.62%) | 2,050,332 (86.15%)  195,852 (8.23%)  133,844 (5.62%) | < 0.001 |
| History of SARS-CoV-2 infection (n, %) | 2,297,185 (77.22%) | 459,437 (77.22%) | 1,837,748 (77.22%) | < 0.001 |
| Vaccination types 1^st^ – 2^nd^ (n, %)  None  Only cDNA vaccine  Only mRNA vaccine  Heterologous vaccination | 595,007 (20.00%)  333,698 (11.22%)  1,928,363 (64.82%)  117,967 (3.97%) | 595,007 (100.00%) | 333,698 (14.02%)  1,928,363 (81.02%)  117,967 (4.96%) | N/A |
| Vaccination interval (mean ± SD, days) | 47.82 ± 32.50 | N/A | 47.82± 32.50 | N/A |

n, number; SMD, standard mean difference; SD, standard deviation; SARS-CoV-2; Severe acute respiratory syndrome coronavirus-2.

**3. Table S3:** Baseline characteristics for sub-cohort after 1:2 propensity score matching

**Table S3** Baseline characteristics for the matched population between non–booster and booster group

| **Crude cohort** | **Total**  **(n = 1,067,688)** | **Non–booster group**  **(n = 355,896)** | **Booster group**  **(n = 711,792)** | **SMD** |
| --- | --- | --- | --- | --- |
| Sex (n, %)  Male  Female | 425,055 (39.81%)  642,633 (60.19%) | 141,685 (39.81%)  214,211 (60.19%) | 283,370 (39.81%)  428,422 (60.19%) | < 0.001 |
| Age (mean ± SD, years)  < 65 years (n, %)  65 to 74 years (n, %)  ≥ 75 years (n, %) | 46.19 ± 14.84  367,296 (34.40%)  605,544 (56.72%)  94,848 (8.88%) | 46.19 ± 14.84  122,432 (34.40%)  201,848 (56.72%)  31,616 (8.88%) | 46.19 ± 14.84  244,864 (34.40%)  403,696 (56.72%)  63,232 (8.88%) | < 0.001  < 0.001 |
| Income (n, %)  Low  Middle  High | 263,144 (24.65%)  305,514 (28.61%)  499,030 (46.74%) | 86,029 (24.17%)  97,757 (27.47%)  172,110 (48.36%) | 177,115 (24.88%)  207,757 (29.19%)  326,920 (45.93%) | 0.049 |
| Charlson’s comorbidity index (n, %)  0  1  ≥ 2 | 844,816 (79.13%)  151,158 (14.16%)  71,714 (6.72%) | 281,606 (79.13%)  50,419 (14.17%)  23,871 (6.71%) | 563,210 (79.13%)  100,739 (14.15%)  47,843 (6.72%) | < 0.001 |
| History of SARS-CoV-2 infection (n, %) | 487,447 (45.65%) | 162,892 (45.77%) | 324,555 (45.60%) | < 0.001 |
| Booster vaccination (n, %)  AZD1222  BNT162b2  mRNA-1273 | N/A | N/A | 5 (0.00%)  623,688 (87.62%)  88,100 (12.38%) | N/A |

n, number; SMD, standardized mean difference; SD, standard deviation; SARS-CoV-2; Severe acute respiratory syndrome coronavirus-2; AZD1222, AstraZeneca ChAdOx1 – S recombinant vaccine; BNT162b2, Pfizer – BioNTech Comirnaty; mRNA-1273, Moderna Spikevax.

**4. Table S4:** The data of cumulative incidences on overall cancers.

**Table S4** Cumulative incidences of overall cancers in the matched cohort between vaccinated and unvaccinated individuals

| **Cumulative incidences of overall cancers**  Number: 595,007 in the unvaccinated group and 2,380,028 in the vaccinated group) | | | | | | | | | | | | | | | | |
| --- | --- | --- | --- | --- | --- | --- | --- | --- | --- | --- | --- | --- | --- | --- | --- | --- |
| **V** | | **One month** | | | **Three months** | | | **Six months** | | | **Nine months** | | | **One year** | | |
|  |  | **Event** | **I** | **95% CI** | **Event** | **I** | **95% CI** | **Event** | **I** | **95% CI** | **Event** | **I** | **95% CI** | **Event** | **I** | **95% CI** |
| No | | 97 | 1.63 | 1.31 – 1.95 | 324 | 5.45 | 4.85 – 6.04 | 843 | 14.17 | 13.21 – 15.12 | 1,439 | 24.18 | 22.94 – 25.43 | 1,989 | 33.43 | 31.96 – 34.89 |
| Yes | | 693 | 2.91 | 2.69 – 3.13 | 2,538 | 10.66 | 10.25 – 11.08 | 5,126 | 21.54 | 20.95 – 22.13 | 7,694 | 32.33 | 31.61 – 33.05 | 10,144 | 42.62 | 41.79 – 43.45 |
| **Stratified by vaccine type**  cDNA vaccine: 333,698; mRNA vaccine: 1,928,363; Heterologous vaccination: 117,967 | | | | | | | | | | | | | | | | |
| cDNA vaccine | | 177 | 5.30 | 4.52 – 6.09 | 701 | 21.01 | 19.45 – 22.56 | 1431 | 42.88 | 40.67 – 45.10 | 2108 | 63.17 | 60.48 – 65.86 | 2817 | 84.42 | 81.31 – 87.52 |
| mRNA vaccine | | 479 | 2.48 | 2.26 – 2.71 | 1733 | 8.99 | 8.56 – 9.41 | 3472 | 18.00 | 17.41 – 18.60 | 5245 | 27.20 | 26.46 – 27.93 | 6878 | 35.67 | 34.83 – 36.51 |
| Heterologous | | 37 | 3.14 | 2.13 – 4.15 | 104 | 8.82 | 7.12 – 10.51 | 223 | 18.90 | 16.42 – 21.38 | 341 | 28.91 | 25.84 – 31.97 | 449 | 38.06 | 34.55 – 41.58 |
| **Cumulative incidences of overall cancers stratified by sex**  Males: 277,149 in the unvaccinated group and 1,108,596 in the vaccinated group.  Females: 317,858 in the unvaccinated group and 1,271,432 in the vaccinated group. | | | | | | | | | | | | | | | | |
| **V** | **Sex** | **One month** | | | **Three months** | | | **Six months** | | | **Nine months** | | | **One year** | | |
|  |  | **Event** | **I** | **95% CI** | **Event** | **I** | **95% CI** | **Event** | **I** | **95% CI** | **Event** | **I** | **95% CI** | **Event** | **I** | **95% CI** |
| No | Male | 34 | 1.23 | 0.81 – 1.64 | 111 | 4.01 | 3.26 – 4.75 | 302 | 10.90 | 9.67 – 12.12 | 508 | 18.33 | 16.74 – 19.92 | 755 | 27.24 | 25.30 – 29.18 |
| Yes |  | 268 | 2.42 | 2.13 – 2.71 | 970 | 8.75 | 8.20 – 9.30 | 1984 | 17.90 | 17.11 – 18.68 | 3003 | 27.09 | 26.12 – 28.06 | 3990 | 35.99 | 34.88 – 37.11 |
| No | Female | 63 | 1.98 | 1.49 – 2.47 | 213 | 6.70 | 5.80 – 7.60 | 541 | 17.02 | 15.59 – 18.45 | 931 | 29.29 | 27.41 – 31.17 | 1234 | 38.82 | 36.66 – 40.98 |
| Yes |  | 425 | 3.34 | 3.02 – 3.66 | 1568 | 12.33 | 11.72 – 12.94 | 3142 | 24.71 | 23.85 – 25.58 | 4691 | 36.90 | 35.84 – 37.95 | 6154 | 48.40 | 47.20 – 49.61 |
| **Cumulative incidences of overall cancers stratified by age**  Age < 65 years: 522,722 in the unvaccinated group and 2,090,888 in the vaccinated group.  Age 65 – 74 years: 40,213 in the unvaccinated group and 160,852 in the vaccinated group.  Age ≥ 75 years: 32,072 in the unvaccinated group and 128,288 in the vaccinated group. | | | | | | | | | | | | | | | | |
| **V** | **Age** | **One month** | | | **Three months** | | | **Six months** | | | **Nine months** | | | **One year** | | |
|  |  | **Event** | **I** | **95% CI** | **Event** | **I** | **95% CI** | **Event** | **I** | **95% CI** | **Event** | **I** | **95% CI** | **Event** | **I** | **95% CI** |
| No | < 65 | 65 | 1.24 | 0.94 – 1.55 | 233 | 4.46 | 3.89 – 5.03 | 593 | 11.34 | 10.43 – 12.26 | 1008 | 19.28 | 18.09 – 20.47 | 1373 | 26.27 | 24.88 – 27.65 |
| Yes |  | 462 | 2.21 | 2.01 – 2.41 | 1740 | 8.32 | 7.93 – 8.71 | 3470 | 16.60 | 16.04 – 17.15 | 5231 | 25.02 | 24.34 – 25.70 | 6861 | 32.81 | 32.04 – 33.59 |
| No | 65 – 74 | 20 | 4.97 | 2.79 – 7.15 | 55 | 13.68 | 10.07 – 17.29 | 160 | 39.79 | 33.64 – 45.94 | 260 | 64.66 | 56.82 – 72.49 | 358 | 89.03 | 79.85 – 98.21 |
| Yes |  | 112 | 6.96 | 5.67 – 8.25 | 427 | 26.55 | 24.03 – 29.06 | 875 | 54.40 | 50.80 – 57.99 | 1307 | 81.25 | 76.87 – 85.64 | 1744 | 108.42 | 103.36 – 113.48 |
| No | ≥ 75 | 12 | 3.74 | 1.63 – 5.86 | 36 | 11.22 | 7.56 – 14.89 | 90 | 28.06 | 22.27 – 33.85 | 171 | 53.32 | 45.35 – 61.29 | 258 | 80.44 | 70.67 – 90.22 |
| Yes |  | 119 | 9.28 | 7.61 – 10.94 | 371 | 28.92 | 25.98 – 31.86 | 781 | 60.88 | 56.62 – 65.14 | 1156 | 90.11 | 84.94 – 95.28 | 1539 | 119.96 | 114.01 – 125.92 |

Cumulative incidence was presented by incidences per 10,000 individuals. V, vaccination; I, cumulative incidence.
