# Supplementary material for: 1-year risks of cancers associated with COVID-19 vaccination: a large population-based cohort study in South Korea
Source: Biomark Res. 2025 Sep 26;13:114. doi: 10.1186/s40364-025-00831-w (PMC12465339; doi:10.1186/s40364-025-00831-w)
Supplement: Supplementary file 2 — Supplementary Material 2 [file 40364_2025_831_MOESM2_ESM.docx]

**Additional File 1 : Detailed method for**

**“1-year risks of cancers associated with COVID-19 vaccination: A large population-based cohort study in South Korea “**

- List-

1. Detailed Method for this study

2. Figure S1: Study flowchart.

3. Table S1: Overview of study description for ICD-10 codes

**1. Detailed Methods**

**Data source**

A population-based retrospective study was designed using the Korean National Health Insurance (KNHI) database, which includes the medical and surgical histories of patients covered by health insurance, representing 98% of the South Korean population [1]. The registered data were coded using the International Classification of Diseases, Tenth Revision (ICD-10), and extracted based on the primary diagnosis during the observation period between January 1, 2021, and December 31, 2023. The study was performed in accordance with the Declaration of Helsinki and the Strengthening the Reporting of Observational Studies in Epidemiology (STROBE) guidelines [2]. The study protocol was approved by our institutional review board, and a waiver of informed consent was obtained. Data were retrospectively analyzed using anonymized personal information from the KNHI database.

**Study cohort**

A total of 8,407,849 individuals aged 20 years or older and residing in Seoul, South Korea, were initially screened and categorized by COVID-19 vaccination status: unvaccinated individuals (n = 679,479) and vaccinated individuals (n = 7,728,370). For the unvaccinated group, the index date was set as January 1, 2022; those who had a vaccination history within 1 year based on the index date and those who were deceased were excluded (n = 30,955). A total of 599,124 unvaccinated individuals were included. For the vaccinated group, the index date was set as the day after the vaccination completion date, and those with incomplete vaccination (n = 278,610), unspecified vaccine type (n = 77,674), deceased (n = 33,836), and a prior medical history of overall cancers within 1-year based on index date (n = 499,572) were excluded. A total of 6,826,856 vaccinated individuals were included. After the 1:4 propensity score matching (PSM) to balance the distribution of covariates between the unvaccinated and vaccinated individuals. Finally, 595,007 and 2,380,028 individuals were included in the unvaccinated and vaccinated groups, respectively (**Figure S1A**).

The index date was set as the second vaccination + 90 days and the day after the third vaccination to evaluate the effect of booster dose (the third vaccination). Since booster doses were defined as the third vaccination, only three COVID-19 vaccines were included in this vaccinated cohort: AZD1222, BNT162b2, and mRNA–1273. After individuals were excluded based on the index date, the vaccinated cohort for booster dose evaluation was finally included (n = 1,867,320) and then divided into the non-booster individuals (the individuals who received up to the second vaccination, n = 388,373) and the booster individuals (the individuals who received the third vaccination, n = 1,478,947). After the 1:2 PSM to balance the distribution of covariates between the two groups, 355,896 and 711,792 individuals were included in the non-booster and booster groups, respectively (**Figure S1B**).

**Exposures and outcomes**

The primary outcomes of this study were cumulative incidences of cancer at 1 month, 3 months, 6 months, 9 months, and 1 year following COVID-19 vaccination. The primary outcomes included the 1-year subsequent risks of cancer by COVID-19 vaccination. The secondary outcomes included the 1-year subsequent risks of overall cancers according to the types of COVID-19 vaccines and the cumulative incidences of overall cancers according to gender (male and female) and age (<65 years, 65–74 years, and ≥75 years) stratifications.

Our target cancers considered the overall (except non-melanoma skin cancer), brain, oral, salivary, oropharynx, larynx, thyroid, esophagus, gastric, colorectal, liver, gallbladder, cholangiocarcinoma, pancreatic, lung, kidney, bladder, breast, vulvar, cervical, uterine, ovarian, testicular, prostate, melanoma, Kaposi’s sarcoma, Hodgkin lymphoma, non-Hodgkin lymphoma, myeloma, and leukemia [3,4]. The list of all cancer types, including corresponding ICD-10 codes, is presented in **Table S1**. Three COVID-19 vaccine types were included: mRNA-based vaccine only (only mRNA vaccine), adenoviral-based vaccine only (only cDNA vaccine), and cross-vaccination between first and second vaccinations (heterologous vaccination).

**Covariates**

The following covariates were considered: age, sex, insurance levels, Charlson comorbidity index (CCI) scores, and prior COVID-19 infection (history of SARS-CoV-2 infection). Insurance levels (recipients of medical aid, grades 1–5, grades 6–10, grades 11–15, and grades 16–20) were defined based on the National Health Insurance premium, which was used as a proxy for income since it is proportional to monthly income and includes both earnings and capital gains. The presence of CCI conditions and COVID-19 infection was determined by the existence of a primary or secondary diagnosis recorded at least twice within 1 year before the index date [5].

**Statistical analysis**

Data were statistically analyzed using the SAS Enterprise Guide (version 8.3., SAS Institute, Cary, NC, USA) and visualized using Python (version 3.11.5., Python Software Foundation, Wilmington, DE, USA) with *matplotlib* (version 3.7.2.). Since most individuals received the COVID-19 vaccination, the imbalance between groups was considered using the PSM. The method adopted for PSM was the greedy nearest neighbor matching without replacement by using a specified caliper width of 0.001 standard deviation (SD). The adequacy of PSM was evaluated using standardized mean differences (SMDs), where SMD < 0.1 indicated no significant imbalances between the two cohorts. A normal distribution was confirmed with the Kolmogorov–Smirnov test. Data were reported as means ± SD for continuous variables and as numbers and percentages for categorical variables. The cumulative incidences of all cancer types were calculated with cumulative incidences per 10,000 individuals with 95% confidence intervals (CIs), which were compared between the unvaccinated and vaccinated groups. A multivariate Cox proportional hazard regression model considering confounding factors was used to estimate the risks of all cancer types, presenting the hazard ratios (HRs) and 95% CIs. Two-sided *P*-values of <0.05 were considered to indicate statistical significance.

**Limitations of this study**

This study has several limitations. Although our data provide insights into COVID-19 vaccination–associated cancer risk, our findings do not establish causal relationships. Furthermore, as most solid tumours require more than 1 year to develop, our one-year follow-up period is relatively short for evaluating cancer incidence, and the possibility of reverse causation or surveillance bias cannot be excluded. For the surveillance issue, there may be systematic differences in health surveillance intensity between the two groups. However, given South Korea’s single-payer healthcare system and its high accessibility, individuals generally have a uniformly high rate of medical center visits, which may reduce such differences. In addition, the study region (Seoul) is considered one of the most medically accessible areas, further minimizing spatial or socioeconomic inequalities. Therefore, cautious interpretation of our findings is warranted. Despite these limitations, our analysis provides valuable insight regarding potential cancer risks and their associations with COVID-19 vaccination.

**References**

1. Oh J, Lee M, Kim M, Kim HJ, Lee SW, Rhee SY, et al. Incident allergic diseases in post-COVID-19 condition: multinational cohort studies from South Korea, Japan and the UK. Nat Commun. 2024;15:2830.

2. von Elm E, Altman DG, Egger M, Pocock SJ, Gøtzsche PC, Vandenbroucke JP. Strengthening the Reporting of Observational Studies in Epidemiology (STROBE) statement: guidelines for reporting observational studies. Bmj. 2007;335:806-8.

3. Siegel RL, Miller KD, Wagle NS, Jemal A. Cancer statistics, 2023. CA Cancer J Clin. 2023;73:17-48.

4. Sung H, Ferlay J, Siegel RL, Laversanne M, Soerjomataram I, Jemal A, et al. Global Cancer Statistics 2020: GLOBOCAN Estimates of Incidence and Mortality Worldwide for 36 Cancers in 185 Countries. CA Cancer J Clin. 2021;71:209-49.

5. Sundararajan V, Henderson T, Perry C, Muggivan A, Quan H, Ghali WA. New ICD-10 version of the Charlson comorbidity index predicted in-hospital mortality. J Clin Epidemiol. 2004;57:1288-94.

**2. Figure S1:** Study flowchart.

**
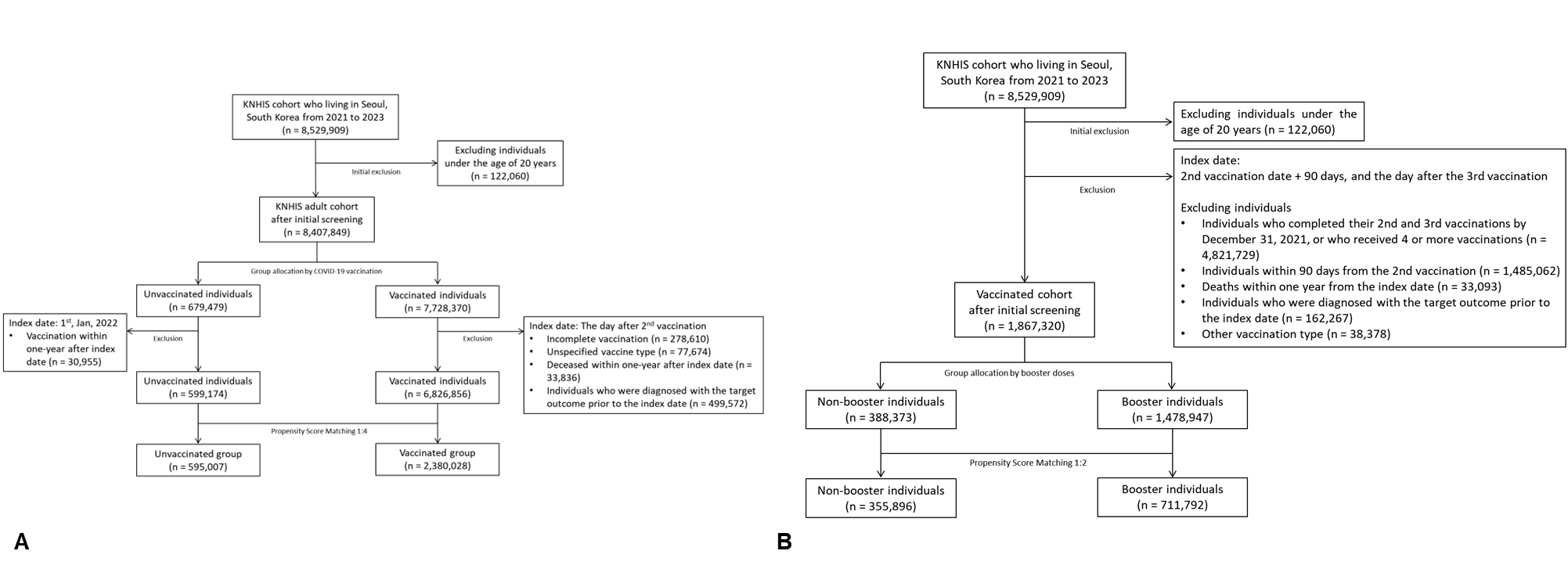
**

**Figure S1**. **Study flowchart**. (A) Main cohort to compare the effect of COVID-19 vaccination, (B) Vaccinated cohort to compare the effect of booster dose of COVID-19 vaccine.

**3. Table S1:** Overview of study description for ICD-10 codes

**Table S1** Overview of study description for cancers after COVID-19 vaccination

| # ICD – 10 codes for adverse events of interest in cancers | | | | |
| --- | --- | --- | --- | --- |
| ***Cancers*** | | ***ICD – 10 codes*** | ***Confounding factors*** | ***ICD – 10 cods*** |
| Overall | Overall cancers | C00 – C96, except for C44 | History of SARS-CoV-2 infection | U071 |
| Brain | Brain cancer | C70 – C72 | Charlson Comorbidity Index | B20 – B24, C0 – C3, C40, C41, C43, C45 – C49, C5, C6, C70 – C85, C91 – C93, C95 – C97, C883, C887, C889, C900, C901, C940 – C943, C947, E10, E11, E13, E14, E102 – E104, E112 – E114, E132 – E134, E142 – E144, F00 – F02, F051, G81, G46, G450 – G452, G454, G458, G459, G820 – G822, I21, I22, I50, I60 – I66, I69, I71, I252, I670 – I679, I681, I682, I688, I739, I790, J40 – J47, J60 – 67, K25 – K28, K73, K702, K703, K717, K721, K729, K740, K742 – K746, K766, K767, M05, M060, M063, M069, M32, M34, M332, M353, N01, N03, N052 – N056, N072 – N074, N18, N19, N25, R02, Z958, Z959 |
| Throat | Oral cancer | C00 – C06 |  |  |
|  | Salivary cancer | C07, C08 |  |  |
|  | Oropharynx cancer | C09, C10 |  |  |
|  | Larynx cancer | C32 |  |  |
|  | Thyroid cancer | C73 |  |  |
| Gastrointestinal system | Esophagus cancer | C15 |  |  |
|  | Gastric cancer | C16 |  |  |
|  | Colorectal cancer | C18 – C20 |  |  |
|  | Liver cancer | C22 |  |  |
|  | Gallbladder cancer | C23 |  |  |
|  | Cholangiocarcinoma | C22.1, C24.0 |  |  |
|  | Pancreatic cancer | C25 |  |  |
| Respiratory system | Lung cancer | C33, C34 |  |  |
| Renal system | Kidney cancer | C64 |  |  |
|  | Bladder cancer | C67 |  |  |
| Female – specific | Breast cancer | C50 | Socioeconomic confounders | Insurance level in KNHI system  (low, middle, and high levels) |
|  | Vulvar cancer | C51 |  |  |
|  | Cervical cancer | C53 | ICD – 10 codes ending with an ‘x’ denote that all subgroups are included.  ICD, international classification of disease; COVID-19, coronavirus-2019. | |
|  | Uterine cancer | C54 |  |  |
|  | Ovarian cancer | C56 |  |  |
| Male – specific | Testicular cancer | C62 |  |  |
|  | Prostate cancer | C61 |  |  |
| Skin, mucous,  and soft – tissue | Melanoma | C43 |  |  |
|  | Kaposi’s sarcoma | C46 |  |  |
| Hematologic system | Hodgkin lymphoma | C81 |  |  |
|  | Non – Hodgkin lymphoma | C82 – C88 |  |  |
|  | Myeloma | C90 |  |  |
|  | Leukemia | C91 – C95 |  |  |
