# Supplementary material for: 1-year risks of cancers associated with COVID-19 vaccination: a large population-based cohort study in South Korea
Source: Biomark Res. 2025 Sep 26;13:114. doi: 10.1186/s40364-025-00831-w (PMC12465339; doi:10.1186/s40364-025-00831-w)
Supplement: Supplementary file 3 — Supplementary Material 3 [file 40364_2025_831_MOESM3_ESM.docx]

**Additional File 3 : Detailed results of specific cancers for**

**“1-year risks of cancers associated with COVID-19 vaccination: A large population-based cohort study in South Korea “**

- List-

1. Detailed results of specific cancers for this study

2. Figure S2: Risks of thyroid cancers associated with COVID-19 vaccines.

3. Table S5: The data of cumulative incidences on thyroid cancers.

4. Figure S3: Risks of gastric and colorectal cancers associated with COVID-19 vaccines.

5. Table S6: The detailed cumulative incidence on gastric cancer

6. Table S7: The detailed cumulative incidence on colorectal cancer

7. Figure S4: Risks of lung cancers associated with COVID-19 vaccines.

8. Table S8: The detailed cumulative incidence on lung cancer

9. Figure S5: Risks of breast and prostate cancers associated with COVID-19 vaccines.

10. Table S9: The detailed cumulative incidence on breast cancer

11. Table S10: The detailed cumulative incidence on prostate cancer

**1. Detailed Results**

**Thyroid cancer**

The cumulative incidence of thyroid cancer was significantly higher in the vaccinated group than in the unvaccinated group at 1 month (0.60 vs. 0.27), 3 months (2.22 vs. 0.86), 6 months (4.26 vs. 2.34), 9 months (6.36 vs. 4.52), and 1 year (8.07 vs. 5.90) post-vaccination (**Figure S2A**). All types of COVID-19 vaccines were associated with a significantly increased risk of thyroid cancer 1 year after vaccination. The highest HR was observed in the heterologous vaccination group (HR, 1.70; 95% CI, 1.34–2.09), followed by the mRNA vaccine group (HR, 1.34; 95% CI, 1.19–1.50) and the cDNA vaccine group (HR, 1.29; 95% CI, 1.090–1.53; **Figure S2B**). The 1-year cumulative incidence of thyroid cancer was the highest in vaccinated females (11.69; 95% CI, 11.09–12.28), with statistical significance according to vaccination status (**Figure S2C**). When stratified by age, the cumulative incidence was the highest in the vaccinated individuals aged <65 years (8.46; 95% CI, 8.07–8.85). The cumulative incidence was not significantly different in the vaccinated individuals aged 65–74 years, but it significantly differed in the vaccinated individuals aged <65 and ≥75 years (**Figure S2D**). The detailed cumulative incidence on thyroid cancer are provided in **Table S5**.

**Gastric cancer**

The cumulative incidence of gastric cancer was significantly higher in the vaccinated group than in the unvaccinated group at 1 month (0.32 vs. 0.12), 3 months (1.05 vs. 0.34), 6 months (1.97 vs. 0.94), 9 months (2.84 vs. 1.83), and 1 year (3.70 vs. 2.77) post-vaccination (**Figure S3A**). Among the vaccination types, only cDNA vaccination (HR, 1.92; 95% CI, 1.60–2.31) significantly associated with increased the risk of gastric cancer 1 year after COVID-19 vaccination (**Figure S3B**). The cumulative incidence of gastric cancers did not significantly differ between the vaccinated and unvaccinated females. However, the cumulative incidence of gastric cancers in males was significantly higher in the vaccinated group (4.76; 95% CI, 4.36–5.17) than in the unvaccinated group (3.36; 95% CI, 2.67–4.04; **Figure S3C**). The cumulative incidence did not significantly vary in the vaccinated individuals aged ≥75 years but significantly differed in the vaccinated individuals aged <65 and 65–74 years (**Figure S3D**). The detailed cumulative incidence on gastric cancer are provided in **Table S6**.

**Colorectal cancer**

The cumulative incidence of colorectal cancer was significantly higher in the vaccinated group than in the unvaccinated group at 3 months (1.47 vs. 0.79), 6 months (2.92 vs. 1.90), 9 months (4.24 vs. 3.06), and 1 year (5.46 vs. 4.27) post-vaccination (**Figure S3D**). Among the vaccination types, only cDNA vaccination (HR, 1.45; 95% CI, 1.23–1.69) and only mRNA vaccination (HR, 1.23; 95% CI, 1.07–1.41) significantly associated with increased the risk of gastric cancer 1 year after COVID-19 vaccination (**Figure S3E**). In contrast to the observation in gastric cancer, the cumulative incidence of colorectal cancers in females was significantly higher in the vaccinated group (4.76; 95% CI, 4.38–5.14) than in the unvaccinated group (3.33; 95% CI, 2.70–3.97; **Figure S3F**). The cumulative incidence significantly differed in the vaccinated and unvaccinated individuals aged <65 (**Figure S3G**). The detailed data on colorectal cancer are provided in **Table S7**.

**Lung cancer**

The cumulative incidence of lung cancer was significantly higher in the vaccinated group than in the unvaccinated group after one-month post-vaccination (**Figure S4A**). Among the vaccination types, only cDNA vaccination (HR, 2.22; 95% CI, 1.77–2.74) and only mRNA vaccination (HR, 1.25; 95% CI, 1.01–1.54) significantly associated with increased the risk of lung cancer 1 year after COVID-19 vaccination (**Figure S4B**). The cumulative incidence of lung cancers in males and females was significantly higher in the vaccinated group than in the unvaccinated group. The highest level (3.44; 95% CI, 3.09–3.78) was observed in vaccinated males at 1-year post-vaccination (**Figure S4C**). This parameter significantly differed in the vaccinated and unvaccinated individuals aged <75 years (**Figure S4D**). The detailed data on lung cancer are provided in **Table S8**.

**Breast cancer**

The cumulative incidence of breast cancer was significantly higher in the vaccinated group than in the unvaccinated group at 1 month post-vaccination (**Figure S5A**). The vaccination types except cDNA vaccines significantly associated with increased the risks of breast cancer; the highest risk was observed in heterologous vaccination (HR, 1.67; 95% CI, 1.36–2.05; **Figure S5B**). Significant differences of cumulative incidence were found in the vaccinated and unvaccinated individuals aged <65 years (13.57 in the vaccinated group vs. 10.76 in the unvaccinated group; **Figure S5C**). The detailed data on lung cancer are provided in **Table S9**.

**Prostate cancer**

The cumulative incidence of prostate cancer was significantly higher in the vaccinated group compared to the unvaccinated group after 3 months post-vaccination (**Figure S5D**). Among the vaccination types, only cDNA vaccination (HR, 2.69; 95% CI, 2.12–3.43) significantly associated with increased the risk of prostate cancer 1 year after COVID-19 vaccination (**Figure S5E**). Statistically significant differences of cumulative incidence were found in those aged < 65 years and ≥ 75 years (**Figure S5F**). Detailed data on lung cancer are provided in **Table S10**.

**2. Figure S2:** Risks of thyroid cancers associated with COVID-19 vaccines.


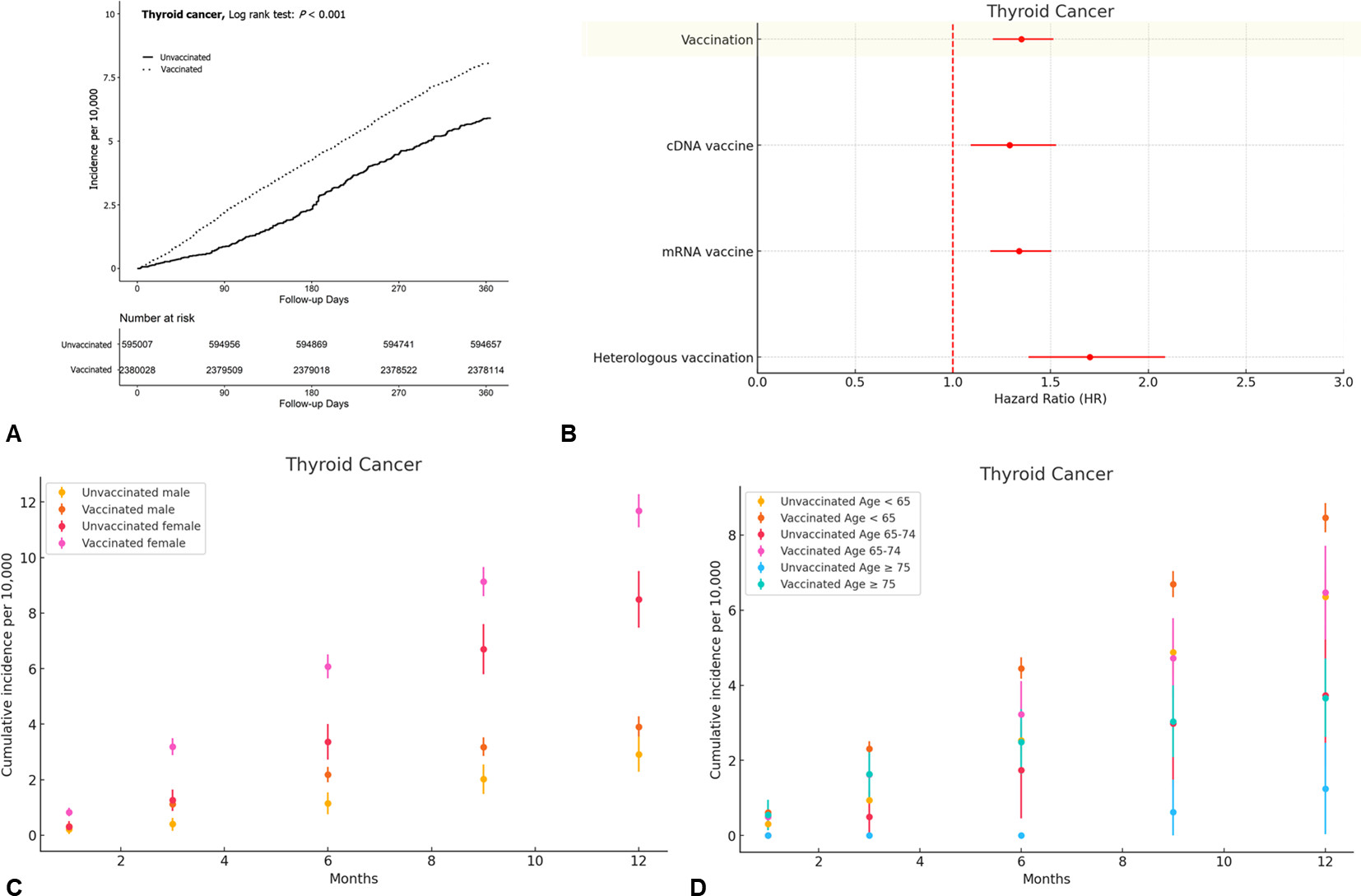


**Figure S2**. **Risks of thyroid cancers associated with COVID-19 vaccines**. (A) Cumulative incidences of thyroid cancers; (B) Hazard ratio of thyroid cancers according to the vaccine types; (D) Cumulative incidences of thyroid cancers stratified by sex; (E) Cumulative incidences of thyroid cancers stratified by age.

**3.** **Table S5:** The data of cumulative incidences on thyroid cancers.

**Table S5** Cumulative incidences of thyroid cancers in the matched cohort between vaccinated and unvaccinated individuals

| **Cumulative incidences of thyroid cancers**  Number: 595,007 in the unvaccinated group and 2,380,028 in the vaccinated group | | | | | | | | | | | | | | | | |
| --- | --- | --- | --- | --- | --- | --- | --- | --- | --- | --- | --- | --- | --- | --- | --- | --- |
| **V** | | **One month** | | | **Three months** | | | **Six months** | | | **Nine months** | | | **One year** | | |
|  |  | **Event** | **I** | **95% CI** | **Event** | **I** | **95% CI** | **Event** | **I** | **95% CI** | **Event** | **I** | **95% CI** | **Event** | **I** | **95% CI** |
| No | | 16 | 0.27 | 0.14 – 0.40 | 51 | 0.86 | 0.62 – 1.09 | 139 | 2.34 | 1.95 – 2.72 | 269 | 4.52 | 3.98 – 5.06 | 351 | 5.90 | 5.28 – 6.52 |
| Yes | | 142 | 0.60 | 0.50 – 0.69 | 529 | 2.22 | 2.03 – 2.41 | 1,015 | 4.26 | 4.00 – 4.53 | 1,514 | 6.36 | 6.04 – 6.68 | 1,920 | 8.07 | 7.71 – 8.43 |
| **Stratified by vaccine type**  cDNA vaccine: 333,698; mRNA vaccine: 1,928,363; Heterologous vaccination: 117,967 | | | | | | | | | | | | | | | | |
| cDNA vaccine | | 23 | 0.69 | 0.41 – 0.97 | 73 | 2.19 | 1.69 – 2.69 | 143 | 4.29 | 3.58 – 4.99 | 197 | 5.90 | 5.08 – 6.73 | 253 | 7.58 | 6.65 – 8.52 |
| mRNA vaccine | | 113 | 0.59 | 0.48 – 0.69 | 429 | 2.22 | 2.01 – 2.44 | 811 | 4.21 | 3.92 – 4.50 | 1,219 | 6.32 | 5.97 – 6.68 | 1,541 | 7.99 | 7.59 – 8.39 |
| Heterologous | | 6 | 0.51 | 0.10 – 0.92 | 27 | 2.29 | 1.43 – 3.15 | 61 | 5.17 | 3.87 – 6.47 | 98 | 8.31 | 6.66 – 9.95 | 126 | 10.68 | 8.82 – 12.54 |
| **Cumulative incidences of thyroid cancers stratified by sex**  Males: 277,149 in the unvaccinated group and 1,108,596 in the vaccinated group.  Females: 317,858 in the unvaccinated group and 1,271,432 in the vaccinated group. | | | | | | | | | | | | | | | | |
| **V** | **Sex** | **One month** | | | **Three months** | | | **Six months** | | | **Nine months** | | | **One year** | | |
|  |  | **Event** | **I** | **95% CI** | **Event** | **I** | **95% CI** | **Event** | **I** | **95% CI** | **Event** | **I** | **95% CI** | **Event** | **I** | **95% CI** |
| No | Male | 6 | 0.22 | 0.04 – 0.39 | 11 | 0.40 | 0.16 – 0.63 | 32 | 1.15 | 0.75 – 1.55 | 56 | 2.02 | 1.49 – 2.55 | 81 | 2.92 | 2.29 – 3.56 |
| Yes |  | 36 | 0.32 | 0.22 – 0.43 | 124 | 1.12 | 0.92 – 1.32 | 242 | 2.18 | 1.91 – 2.46 | 353 | 3.18 | 2.85 – 3.52 | 434 | 3.91 | 3.55 – 4.28 |
| No | Female | 10 | 0.31 | 0.12 – 0.51 | 40 | 1.26 | 0.87 – 1.65 | 107 | 3.37 | 2.73 – 4.00 | 213 | 6.70 | 5.80 – 7.60 | 270 | 8.49 | 7.48 – 9.51 |
| Yes |  | 106 | 0.83 | 0.68 – 0.99 | 405 | 3.19 | 2.88 – 3.50 | 773 | 6.08 | 5.65 – 6.51 | 1161 | 9.13 | 8.61 – 9.66 | 1486 | 11.69 | 11.09 – 12.28 |
| **Cumulative incidences of thyroid cancers stratified by age**  Age < 65 years: 522,722 in the unvaccinated group and 2,090,888 in the vaccinated group.  Age 65 – 74 years: 40,213 in the unvaccinated group and 160,852 in the vaccinated group.  Age ≥ 75 years: 32,072 in the unvaccinated group and 128,288 in the vaccinated group. | | | | | | | | | | | | | | | | |
| **V** | **Age** | **One month** | | | **Three months** | | | **Six months** | | | **Nine months** | | | **One year** | | |
|  |  | **Event** | **I** | **95% CI** | **Event** | **I** | **95% CI** | **Event** | **I** | **95% CI** | **Event** | **I** | **95% CI** | **Event** | **I** | **95% CI** |
| No | < 65 | 16 | 0.31 | 0.16 – 0.46 | 49 | 0.94 | 0.67 – 1.20 | 132 | 2.53 | 2.09 – 2.96 | 255 | 4.88 | 4.28 – 5.48 | 332 | 6.35 | 5.67 – 7.03 |
| Yes |  | 127 | 0.61 | 0.50 – 0.71 | 482 | 2.31 | 2.10 – 2.51 | 931 | 4.45 | 4.17 – 4.74 | 1399 | 6.69 | 6.34 – 7.04 | 1769 | 8.46 | 8.07 – 8.85 |
| No | 65 – 74 | 0 | 0.00 | 0.00 – 0.00 | 2 | 0.50 | 0.00 – 1.19 | 7 | 1.74 | 0.45 – 3.03 | 12 | 2.98 | 1.30 – 4.67 | 15 | 3.73 | 1.84 – 5.62 |
| Yes |  | 8 | 0.50 | 0.15 – 0.84 | 26 | 1.62 | 1.00 – 2.24 | 52 | 3.23 | 2.35 – 4.11 | 76 | 4.72 | 3.66 – 5.79 | 104 | 6.47 | 5.22 – 7.71 |
| No | ≥ 75 | 0 | 0.00 | 0.00 – 0.00 | 0 | 0.00 | 0.00 – 0.00 | 0 | 0.00 | 0.00 – 0.00 | 2 | 0.62 | 0.00 – 1.49 | 4 | 1.25 | 0.03 – 2.47 |
| Yes |  | 7 | 0.55 | 0.14 – 0.95 | 21 | 1.64 | 0.94 – 2.34 | 32 | 2.49 | 1.63 – 3.36 | 39 | 3.04 | 2.09 – 3.99 | 47 | 3.66 | 2.62 – 4.71 |

Cumulative incidence was presented by incidences per 10,000 individuals. I, cumulative incidence.

**4. Figure S3:** Risks of gastric and colorectal cancers associated with COVID-19 vaccines.


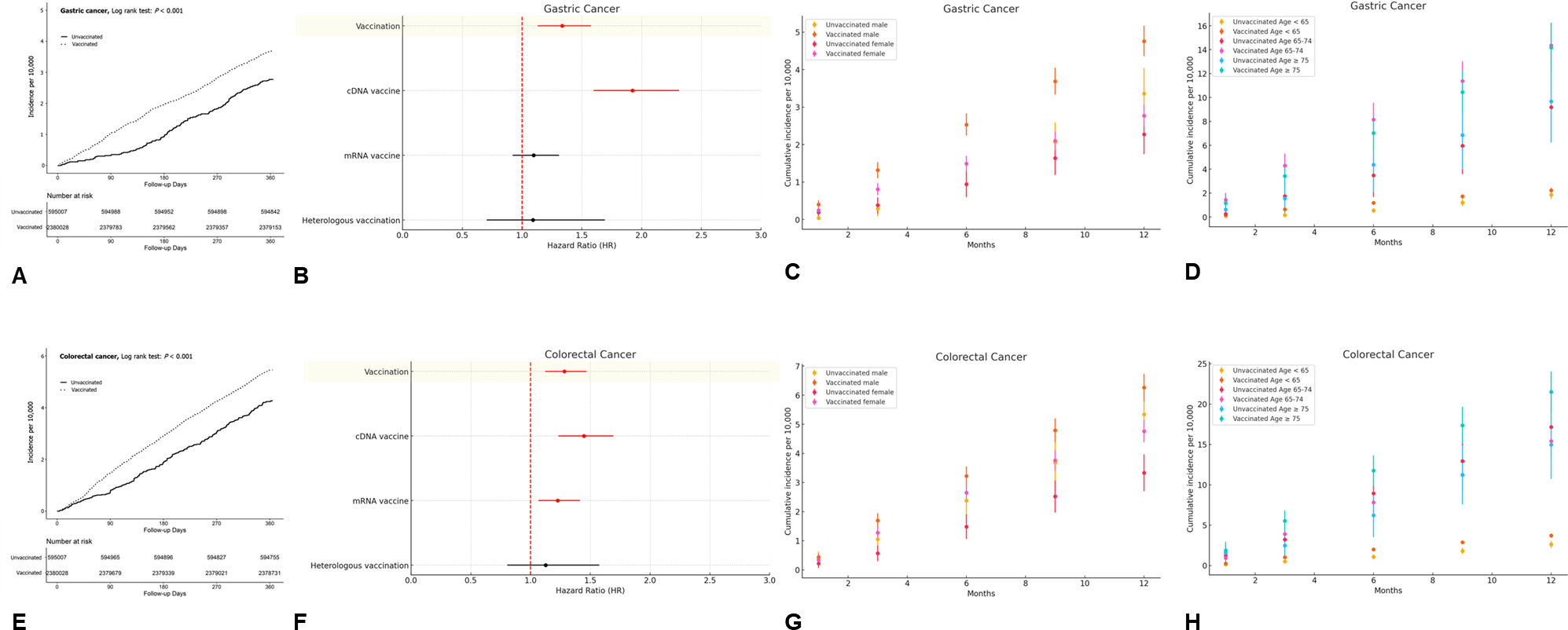


**Figure S3**. **Risks of gastric and colorectal cancers associated with COVID-19 vaccines**. (A) Cumulative incidences of gastric cancers; (B) Hazard ratio of gastric cancers according to the vaccine types; (C) Cumulative incidences of gastric cancers stratified by sex; (D) Cumulative incidences of gastric cancers stratified by age; (E) Cumulative incidences of colorectal cancers; (F) Hazard ratio of colorectal cancers according to the vaccine types; (G) Cumulative incidences of colorectal cancers stratified by sex; (H) Cumulative incidences of colorectal cancers stratified by age.

**5. Table S6:** The detailed cumulative incidence on gastric cancer

**Table S6** Cumulative incidences of gastric cancers in the matched cohort between vaccinated and unvaccinated individuals

| **Cumulative incidences of gastric cancers**  Number: 595,007 in the unvaccinated group and 2,380,028 in the vaccinated group | | | | | | | | | | | | | | | | |
| --- | --- | --- | --- | --- | --- | --- | --- | --- | --- | --- | --- | --- | --- | --- | --- | --- |
| **V** | | **One month** | | | **Three months** | | | **Six months** | | | **Nine months** | | | **One year** | | |
|  |  | **Event** | **I** | **95% CI** | **Event** | **I** | **95% CI** | **Event** | **I** | **95% CI** | **Event** | **I** | **95% CI** | **Event** | **I** | **95% CI** |
| No | | 7 | 0.12 | 0.03 – 0.20 | 20 | 0.34 | 0.19 – 0.48 | 56 | 0.94 | 0.69 – 1.19 | 109 | 1.83 | 1.49 – 2.18 | 165 | 2.77 | 2.35 – 3.20 |
| Yes | | 76 | 0.32 | 0.25 – 0.39 | 249 | 1.05 | 0.92 – 1.18 | 470 | 1.97 | 1.80 – 2.15 | 676 | 2.84 | 2.63 – 3.05 | 880 | 3.70 | 3.45 – 3.94 |
| **Stratified by vaccine type**  cDNA vaccine: 333,698; mRNA vaccine: 1,928,363; Heterologous vaccination: 117,967 | | | | | | | | | | | | | | | | |
| cDNA vaccine | | 29 | 0.87 | 0.55 – 1.19 | 106 | 3.18 | 2.57 – 3.78 | 204 | 6.11 | 5.27 – 6.95 | 288 | 8.63 | 7.63 – 9.63 | 361 | 10.82 | 9.70 – 11.93 |
| mRNA vaccine | | 44 | 0.23 | 0.16 – 0.30 | 140 | 0.73 | 0.61 – 0.85 | 256 | 1.33 | 1.16 – 1.49 | 371 | 1.92 | 1.73 – 2.12 | 496 | 2.57 | 2.35 – 2.80 |
| Heterologous | | 3 | 0.25 | 0.00 – 0.54 | 3 | 0.25 | 0.00 – 0.54 | 10 | 0.85 | 0.32 – 1.37 | 17 | 1.44 | 0.76 – 2.13 | 23 | 1.95 | 1.15 – 2.75 |
| **Cumulative incidences of gastric cancers stratified by sex**  Males: 277,149 in the unvaccinated group and 1,108,596 in the vaccinated group.  Females: 317,858 in the unvaccinated group and 1,271,432 in the vaccinated group. | | | | | | | | | | | | | | | | |
| **V** | **Sex** | **One month** | | | **Three months** | | | **Six months** | | | **Nine months** | | | **One year** | | |
|  |  | **Event** | **I** | **95% CI** | **Event** | **I** | **95% CI** | **Event** | **I** | **95% CI** | **Event** | **I** | **95% CI** | **Event** | **I** | **95% CI** |
| No | Male | 1 | 0.04 | 0.00 – 0.11 | 8 | 0.29 | 0.09 – 0.49 | 26 | 0.94 | 0.58 – 1.30 | 57 | 2.06 | 1.52 – 2.59 | 93 | 3.36 | 2.67 – 4.04 |
| Yes |  | 44 | 0.40 | 0.28 – 0.51 | 146 | 1.32 | 1.10 – 1.53 | 281 | 2.53 | 2.24 – 2.83 | 409 | 3.69 | 3.33 – 4.05 | 528 | 4.76 | 4.36 – 5.17 |
| No | Female | 6 | 0.19 | 0.04 – 0.34 | 12 | 0.38 | 0.16 – 0.59 | 30 | 0.94 | 0.61 – 1.28 | 52 | 1.64 | 1.19 – 2.08 | 72 | 2.27 | 1.74 – 2.79 |
| Yes |  | 32 | 0.25 | 0.16 – 0.34 | 103 | 0.81 | 0.65 – 0.97 | 189 | 1.49 | 1.27 – 1.70 | 267 | 2.10 | 1.85 – 2.35 | 352 | 2.77 | 2.48 – 3.06 |
| **Cumulative incidences of gastric cancers stratified by age**  Age < 65 years: 522,722 in the unvaccinated group and 2,090,888 in the vaccinated group.  Age 65 – 74 years: 40,213 in the unvaccinated group and 160,852 in the vaccinated group.  Age ≥ 75 years: 32,072 in the unvaccinated group and 128,288 in the vaccinated group. | | | | | | | | | | | | | | | | |
| **V** | **Age** | **One month** | | | **Three months** | | | **Six months** | | | **Nine months** | | | **One year** | | |
|  |  | **Event** | **I** | **95% CI** | **Event** | **I** | **95% CI** | **Event** | **I** | **95% CI** | **Event** | **I** | **95% CI** | **Event** | **I** | **95% CI** |
| No | < 65 | 4 | 0.08 | 0.00 – 0.15 | 8 | 0.15 | 0.05 – 0.26 | 28 | 0.54 | 0.34 – 0.73 | 63 | 1.21 | 0.91 – 1.50 | 97 | 1.86 | 1.49 – 2.22 |
| Yes |  | 38 | 0.18 | 0.12 – 0.24 | 136 | 0.65 | 0.54 – 0.76 | 249 | 1.19 | 1.04 – 1.34 | 359 | 1.72 | 1.54 – 1.89 | 467 | 2.23 | 2.03 – 2.44 |
| No | 65 – 74 | 1 | 0.25 | 0.00 – 0.74 | 7 | 1.74 | 0.45 – 3.03 | 14 | 3.48 | 1.66 – 5.30 | 24 | 5.97 | 3.58 – 8.36 | 37 | 9.20 | 6.24 – 12.16 |
| Yes |  | 23 | 1.43 | 0.85 – 2.01 | 69 | 4.29 | 3.28 – 5.30 | 131 | 8.14 | 6.75 – 9.54 | 183 | 11.38 | 9.73 – 13.02 | 231 | 14.36 | 12.51 – 16.21 |
| No | ≥ 75 | 2 | 0.62 | 0.00 – 1.49 | 5 | 1.56 | 0.19 – 2.93 | 14 | 4.37 | 2.08 – 6.65 | 22 | 6.86 | 3.99 – 9.72 | 31 | 9.67 | 6.26 – 13.07 |
| Yes |  | 15 | 1.17 | 0.58 – 1.76 | 44 | 3.43 | 2.42 – 4.44 | 90 | 7.02 | 5.57 – 8.46 | 134 | 10.45 | 8.68 – 12.21 | 182 | 14.19 | 12.13 – 16.25 |

Cumulative incidence was presented by incidences per 10,000 individuals. I, cumulative incidence.

**6. Table S7:** The detailed cumulative incidence on colorectal cancer

**Table S7** Cumulative incidences of colorectal cancers in the matched cohort between vaccinated and unvaccinated individuals

| **Cumulative incidences of colorectal cancers**  Number: 595,007 in the unvaccinated group and 2,380,028 in the vaccinated group | | | | | | | | | | | | | | | | |
| --- | --- | --- | --- | --- | --- | --- | --- | --- | --- | --- | --- | --- | --- | --- | --- | --- |
| **V** | | **One month** | | | **Three months** | | | **Six months** | | | **Nine months** | | | **One year** | | |
|  |  | **Event** | **I** | **95% CI** | **Event** | **I** | **95% CI** | **Event** | **I** | **95% CI** | **Event** | **I** | **95% CI** | **Event** | **I** | **95% CI** |
| No | | 18 | 0.30 | 0.16 – 0.44 | 47 | 0.79 | 0.56 – 1.02 | 113 | 1.90 | 1.55 – 2.25 | 182 | 3.06 | 2.61 – 3.50 | 254 | 4.27 | 3.74 – 4.79 |
| Yes | | 92 | 0.39 | 0.31 – 0.47 | 351 | 1.47 | 1.32 – 1.63 | 694 | 2.92 | 2.70 – 3.13 | 1009 | 4.24 | 3.98 – 4.50 | 1299 | 5.46 | 5.16 – 5.75 |
| **Stratified by vaccine type**  cDNA vaccine: 333,698; mRNA vaccine: 1,928,363; Heterologous vaccination: 117,967 | | | | | | | | | | | | | | | | |
| cDNA vaccine | | 24 | 0.72 | 0.43 – 1.01 | 104 | 3.12 | 2.52 – 3.72 | 210 | 6.29 | 5.44 – 7.14 | 292 | 8.75 | 7.75 – 9.75 | 399 | 11.96 | 10.78 – 13.13 |
| mRNA vaccine | | 65 | 0.34 | 0.26 – 0.42 | 236 | 1.22 | 1.07 – 1.38 | 457 | 2.37 | 2.15 – 2.59 | 681 | 3.53 | 3.27 – 3.80 | 860 | 4.46 | 4.16 – 4.76 |
| Heterologous | | 3 | 0.25 | 0.00 – 0.54 | 11 | 0.93 | 0.38 – 1.48 | 27 | 2.29 | 1.43 – 3.15 | 36 | 3.05 | 2.05 – 4.05 | 40 | 3.39 | 2.34 – 4.44 |
| **Cumulative incidences of colorectal cancers stratified by sex**  Males: 277,149 in the unvaccinated group and 1,108,596 in the vaccinated group.  Females: 317,858 in the unvaccinated group and 1,271,432 in the vaccinated group. | | | | | | | | | | | | | | | | |
| **V** | **Sex** | **One month** | | | **Three months** | | | **Six months** | | | **Nine months** | | | **One year** | | |
|  |  | **Event** | **I** | **95% CI** | **Event** | **I** | **95% CI** | **Event** | **I** | **95% CI** | **Event** | **I** | **95% CI** | **Event** | **I** | **95% CI** |
| No | Male | 11 | 0.40 | 0.16 – 0.63 | 29 | 1.05 | 0.67 – 1.43 | 66 | 2.38 | 1.81 – 2.96 | 102 | 3.68 | 2.97 – 4.39 | 148 | 5.34 | 4.48 – 6.20 |
| Yes |  | 49 | 0.44 | 0.32 – 0.57 | 188 | 1.70 | 1.45 – 1.94 | 357 | 3.22 | 2.89 – 3.55 | 531 | 4.79 | 4.38 – 5.20 | 694 | 6.26 | 5.79 – 6.73 |
| No | Female | 7 | 0.22 | 0.06 – 0.38 | 18 | 0.57 | 0.30 – 0.83 | 47 | 1.48 | 1.06 – 1.90 | 80 | 2.52 | 1.97 – 3.07 | 106 | 3.33 | 2.70 – 3.97 |
| Yes |  | 43 | 0.34 | 0.24 – 0.44 | 163 | 1.28 | 1.09 – 1.48 | 337 | 2.65 | 2.37 – 2.93 | 478 | 3.76 | 3.42 – 4.10 | 605 | 4.76 | 4.38 – 5.14 |
| **Cumulative incidences of colorectal cancers stratified by age**  Age < 65 years: 522,722 in the unvaccinated group and 2,090,888 in the vaccinated group.  Age 65 – 74 years: 40,213 in the unvaccinated group and 160,852 in the vaccinated group.  Age ≥ 75 years: 32,072 in the unvaccinated group and 128,288 in the vaccinated group. | | | | | | | | | | | | | | | | |
| **V** | **Age** | **One month** | | | **Three months** | | | **Six months** | | | **Nine months** | | | **One year** | | |
|  |  | **Event** | **I** | **95% CI** | **Event** | **I** | **95% CI** | **Event** | **I** | **95% CI** | **Event** | **I** | **95% CI** | **Event** | **I** | **95% CI** |
| No | < 65 | 8 | 0.15 | 0.05 – 0.26 | 26 | 0.50 | 0.31 – 0.69 | 57 | 1.09 | 0.81 – 1.37 | 94 | 1.80 | 1.43 – 2.16 | 137 | 2.62 | 2.18 – 3.06 |
| Yes |  | 53 | 0.25 | 0.19 – 0.32 | 217 | 1.04 | 0.90 – 1.18 | 417 | 1.99 | 1.80 – 2.19 | 605 | 2.89 | 2.66 – 3.12 | 775 | 3.71 | 3.45 – 3.97 |
| No | 65 – 74 | 5 | 1.24 | 0.15 – 2.33 | 13 | 3.23 | 1.48 – 4.99 | 36 | 8.95 | 6.03 – 11.88 | 52 | 12.93 | 9.42 – 16.44 | 69 | 17.16 | 13.11 – 21.20 |
| Yes |  | 15 | 0.93 | 0.46 – 1.40 | 63 | 3.92 | 2.95 – 4.88 | 126 | 7.83 | 6.47 – 9.20 | 181 | 11.25 | 9.61 – 12.89 | 248 | 15.42 | 13.50 – 17.34 |
| No | ≥ 75 | 5 | 1.56 | 0.19 – 2.93 | 8 | 2.49 | 0.77 – 4.22 | 20 | 6.24 | 3.50 – 8.97 | 36 | 11.22 | 7.56 – 14.89 | 48 | 14.97 | 10.74 – 19.20 |
| Yes |  | 24 | 1.87 | 1.12 – 2.62 | 71 | 5.53 | 4.25 – 6.82 | 151 | 11.77 | 9.89 – 13.65 | 223 | 17.38 | 15.10 – 19.66 | 276 | 21.51 | 18.98 – 24.05 |

Cumulative incidence was presented by incidences per 10,000 individuals. I, cumulative incidence.

**7. Figure S4:** Risks of lung cancers associated with COVID-19 vaccines.


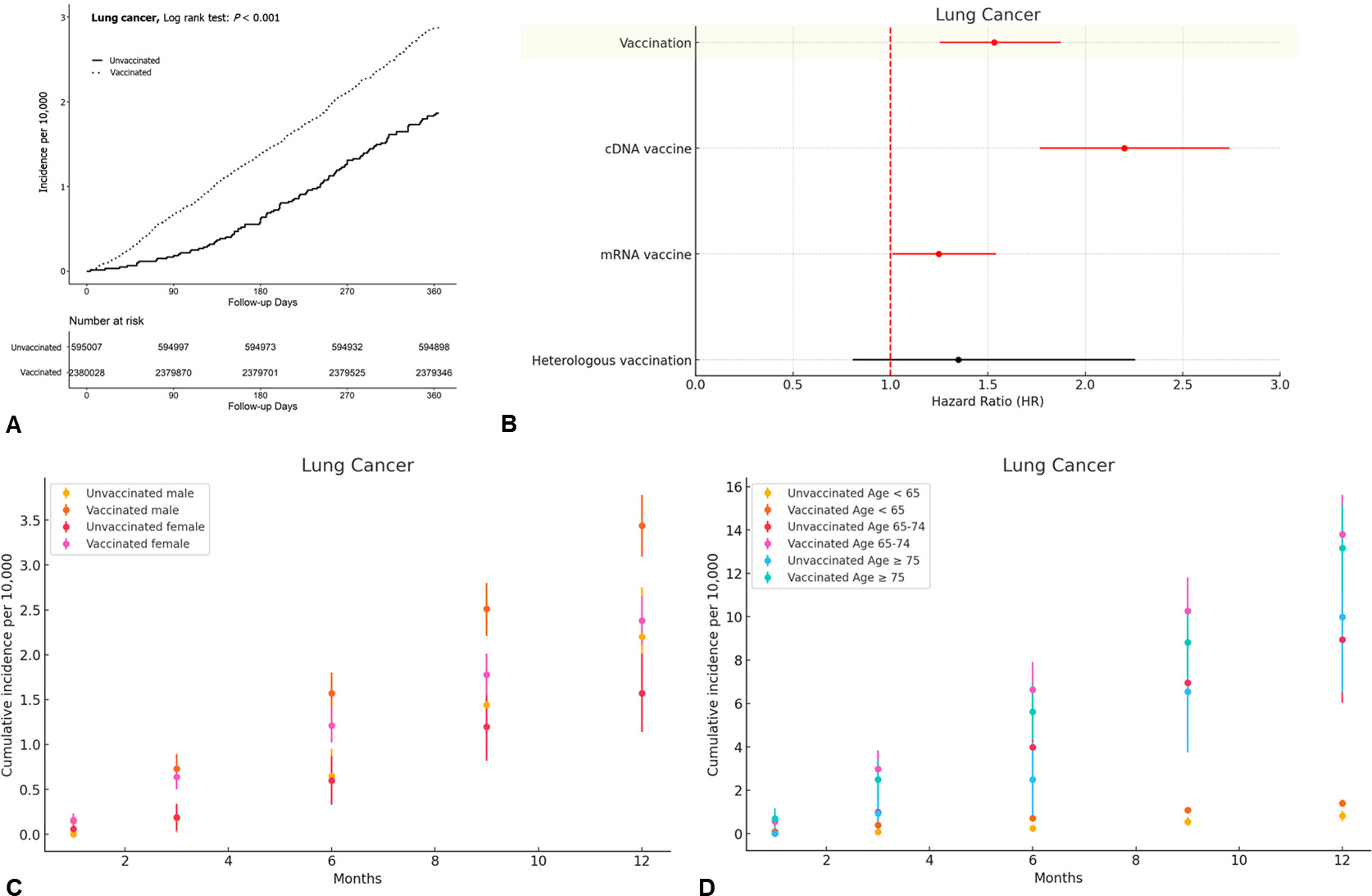


**Figure S4**. **Risks of lung cancers associated with COVID-19 vaccines**. (A) Cumulative incidences of lung cancers; (B) Hazard ratio of lung cancers according to the vaccine types; (C) Cumulative incidences of lung cancers stratified by sex; (D) Cumulative incidences of lung cancers stratified by age.

**8. Table S8:** The detailed cumulative incidence on lung cancer

**Table S8** Cumulative incidences of lung cancers in the matched cohort between vaccinated and unvaccinated individuals

| **Cumulative incidences of lung cancers**  Number: 595,007 in the unvaccinated group and 2,380,028 in the vaccinated group) | | | | | | | | | | | | | | | | |
| --- | --- | --- | --- | --- | --- | --- | --- | --- | --- | --- | --- | --- | --- | --- | --- | --- |
| **V** | | **One month** | | | **Three months** | | | **Six months** | | | **Nine months** | | | **One year** | | |
|  |  | **Event** | **I** | **95% CI** | **Event** | **I** | **95% CI** | **Event** | **I** | **95% CI** | **Event** | **I** | **95% CI** | **Event** | **I** | **95% CI** |
| No | | 2 | 0.03 | 0.00 – 0.08 | 11 | 0.18 | 0.08 – 0.29 | 37 | 0.62 | 0.42 – 0.82 | 78 | 1.31 | 1.02 – 1.60 | 111 | 1.87 | 1.52 – 2.21 |
| Yes | | 37 | 0.16 | 0.11 – 0.21 | 162 | 0.68 | 0.58 – 0.79 | 328 | 1.38 | 1.23 – 1.53 | 504 | 2.12 | 1.93 – 2.30 | 684 | 2.87 | 2.66 – 3.09 |
| **Stratified by vaccine type**  cDNA vaccine: 333,698; mRNA vaccine: 1,928,363; Heterologous vaccination: 117,967 | | | | | | | | | | | | | | | | |
| cDNA vaccine | | 13 | 0.39 | 0.18 – 0.60 | 65 | 1.95 | 1.47 – 2.42 | 135 | 4.05 | 3.36 – 4.73 | 218 | 6.53 | 5.67 – 7.40 | 288 | 8.63 | 7.63 – 9.63 |
| mRNA vaccine | | 23 | 0.12 | 0.07 – 0.17 | 95 | 0.49 | 0.39 – 0.59 | 187 | 0.97 | 0.83 – 1.11 | 274 | 1.42 | 1.25 – 1.59 | 379 | 1.97 | 1.77 – 2.16 |
| Heterologous | | 1 | 0.08 | 0.00 – 0.25 | 2 | 0.17 | 0.00 – 0.40 | 6 | 0.51 | 0.10 – 0.92 | 12 | 1.02 | 0.44 – 1.59 | 17 | 1.44 | 0.76 – 2.13 |
| **Cumulative incidences of lung cancers stratified by sex**  Males: 277,149 in the unvaccinated group and 1,108,596 in the vaccinated group.  Females: 317,858 in the unvaccinated group and 1,271,432 in the vaccinated group. | | | | | | | | | | | | | | | | |
| **V** | **Sex** | **One month** | | | **Three months** | | | **Six months** | | | **Nine months** | | | **One year** | | |
|  |  | **Event** | **I** | **95% CI** | **Event** | **I** | **95% CI** | **Event** | **I** | **95% CI** | **Event** | **I** | **95% CI** | **Event** | **I** | **95% CI** |
| No | Male | 0 | 0.00 | 0.00 – 0.00 | 5 | 0.18 | 0.02 – 0.34 | 18 | 0.65 | 0.35 – 0.95 | 40 | 1.44 | 1.00 – 1.89 | 61 | 2.20 | 1.65 – 2.75 |
| Yes |  | 17 | 0.15 | 0.08 – 0.23 | 81 | 0.73 | 0.57 – 0.89 | 174 | 1.57 | 1.34 – 1.80 | 278 | 2.51 | 2.21 – 2.80 | 381 | 3.44 | 3.09 – 3.78 |
| No | Female | 2 | 0.06 | 0.00 – 0.15 | 6 | 0.19 | 0.04 – 0.34 | 19 | 0.60 | 0.33 – 0.87 | 38 | 1.20 | 0.82 – 1.58 | 50 | 1.57 | 1.14 – 2.01 |
| Yes |  | 20 | 0.16 | 0.09 – 0.23 | 81 | 0.64 | 0.50 – 0.78 | 154 | 1.21 | 1.02 – 1.40 | 226 | 1.78 | 1.55 – 2.01 | 303 | 2.38 | 2.11 – 2.65 |
| **Cumulative incidences of lung cancers stratified by age**  Age < 65 years: 522,722 in the unvaccinated group and 2,090,888 in the vaccinated group.  Age 65 – 74 years: 40,213 in the unvaccinated group and 160,852 in the vaccinated group.  Age ≥ 75 years: 32,072 in the unvaccinated group and 128,288 in the vaccinated group. | | | | | | | | | | | | | | | | |
| **V** | **Age** | **One month** | | | **Three months** | | | **Six months** | | | **Nine months** | | | **One year** | | |
|  |  | **Event** | **I** | **95% CI** | **Event** | **I** | **95% CI** | **Event** | **I** | **95% CI** | **Event** | **I** | **95% CI** | **Event** | **I** | **95% CI** |
| No | < 65 | 2 | 0.04 | 0.00 – 0.09 | 4 | 0.08 | 0.00 – 0.15 | 13 | 0.25 | 0.11 – 0.38 | 29 | 0.55 | 0.35 – 0.76 | 43 | 0.82 | 0.58 – 1.07 |
| Yes |  | 19 | 0.09 | 0.05 – 0.13 | 82 | 0.39 | 0.31 – 0.48 | 149 | 0.71 | 0.60 – 0.83 | 226 | 1.08 | 0.94 – 1.22 | 293 | 1.40 | 1.24 – 1.56 |
| No | 65 – 74 | 0 | 0.00 | 0.00 – 0.00 | 4 | 0.99 | 0.02 – 1.97 | 16 | 3.98 | 2.03 – 5.93 | 28 | 6.96 | 4.38 – 9.54 | 36 | 8.95 | 6.03 – 11.88 |
| Yes |  | 9 | 0.56 | 0.19 – 0.93 | 48 | 2.98 | 2.14 – 3.83 | 107 | 6.65 | 5.39 – 7.91 | 165 | 10.26 | 8.69 – 11.82 | 222 | 13.80 | 11.99 – 15.62 |
| No | ≥ 75 | 0 | 0.00 | 0.00 – 0.00 | 3 | 0.94 | 0.00 – 1.99 | 8 | 2.49 | 0.77 – 4.22 | 21 | 6.55 | 3.75 – 9.35 | 32 | 9.98 | 6.52 – 13.43 |
| Yes |  | 9 | 0.70 | 0.24 – 1.16 | 32 | 2.49 | 1.63 – 3.36 | 72 | 5.61 | 4.32 – 6.91 | 113 | 8.81 | 7.18 – 10.43 | 169 | 13.17 | 11.19 – 15.16 |

Cumulative incidence was presented by incidences per 10,000 individuals. I, cumulative incidence.

**9. Figure S5:** Risks of breast and prostate cancers associated with COVID-19 vaccines.


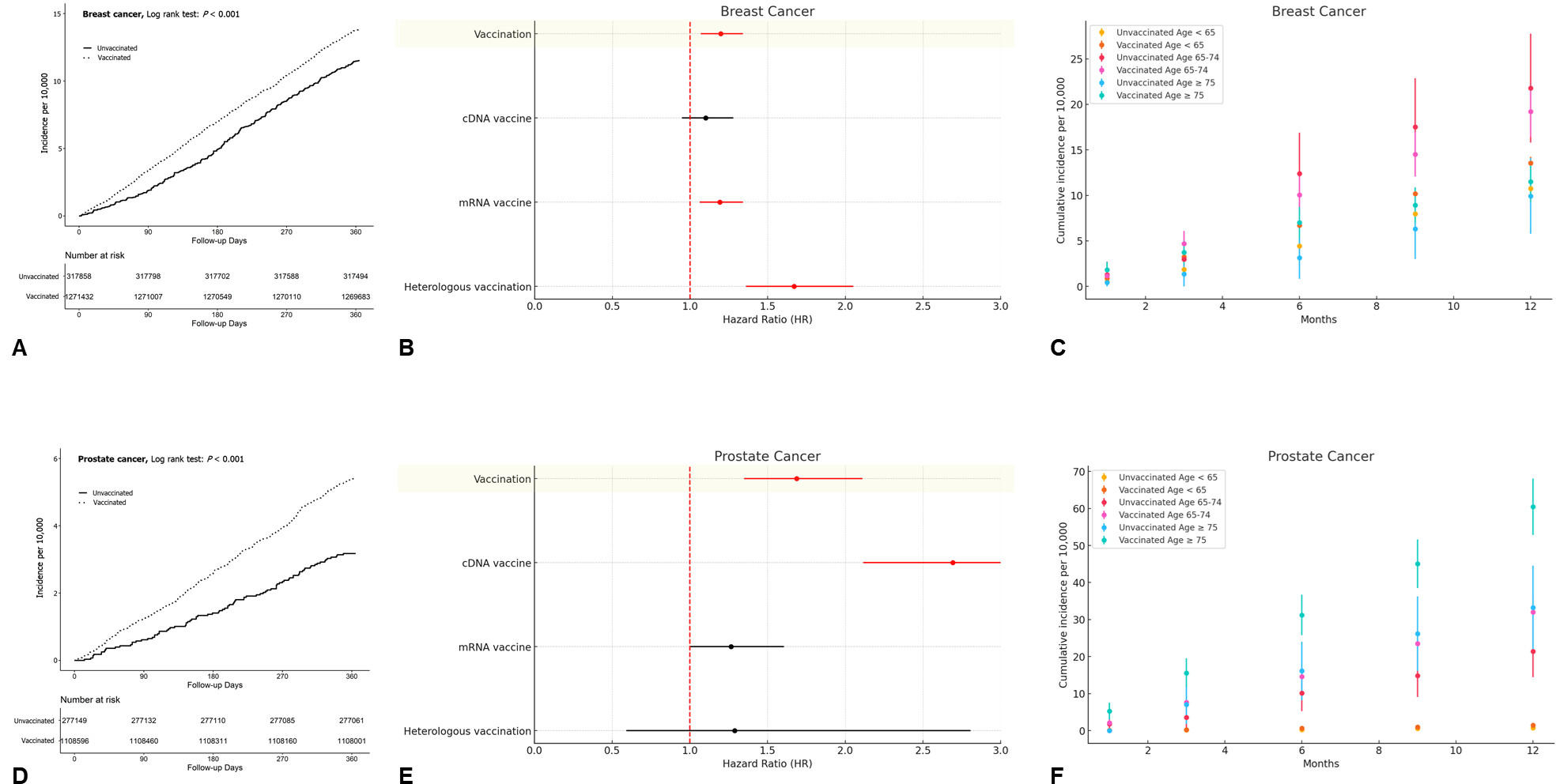


**Figure S5**. **Risks of breast and prostate cancers associated with COVID-19 vaccines**. (A) Cumulative incidences of breast cancers; (B) Hazard ratio of breast cancers according to the vaccine types; (C) Cumulative incidences of lung cancers stratified by age; (D) Cumulative incidences of prostate cancers; (E) Hazard ratio of prostate cancers according to the vaccine types; (F) Cumulative incidences of prostate cancers stratified by age.

**10. Table S9:** The detailed cumulative incidence on breast cancer

**Table S9** Cumulative incidences of breast cancers in the matched cohort between vaccinated and unvaccinated individuals

| **Cumulative incidences of breast cancers**  Number: 595,007 in the unvaccinated group and 2,380,028 in the vaccinated group) | | | | | | | | | | | | | | | | |
| --- | --- | --- | --- | --- | --- | --- | --- | --- | --- | --- | --- | --- | --- | --- | --- | --- |
| **V** | | **One month** | | | **Three months** | | | **Six months** | | | **Nine months** | | | **One year** | | |
|  |  | **Event** | **I** | **95% CI** | **Event** | **I** | **95% CI** | **Event** | **I** | **95% CI** | **Event** | **I** | **95% CI** | **Event** | **I** | **95% CI** |
| No | | 19 | 0.60 | 0.33 – 0.87 | 60 | 1.89 | 1.41 – 2.37 | 157 | 4.94 | 4.17 – 5.71 | 272 | 8.56 | 7.54 – 9.57 | 366 | 11.51 | 10.34 – 12.69 |
| Yes | | 124 | 0.98 | 0.80 – 1.15 | 432 | 3.40 | 3.08 – 3.72 | 889 | 6.99 | 6.53 – 7.45 | 1325 | 10.42 | 9.86 – 10.98 | 1760 | 13.84 | 13.20 – 14.49 |
| **Stratified by vaccine type**  cDNA vaccine: 333,698; mRNA vaccine: 1,928,363; Heterologous vaccination: 117,967 | | | | | | | | | | | | | | | | |
| cDNA vaccine | | 23 | 1.20 | 0.71 – 1.70 | 95 | 4.98 | 3.98 – 5.98 | 179 | 9.38 | 8.00 – 10.75 | 259 | 13.57 | 11.92 – 15.22 | 345 | 18.07 | 16.17 – 19.98 |
| mRNA vaccine | | 84 | 0.83 | 0.65 – 1.01 | 300 | 2.97 | 2.63 – 3.30 | 644 | 6.37 | 5.88 – 6.86 | 976 | 9.65 | 9.05 – 10.26 | 1293 | 12.79 | 12.09 – 13.48 |
| Heterologous | | 17 | 2.45 | 1.29 – 3.61 | 37 | 5.33 | 3.61 – 7.05 | 66 | 9.51 | 7.22 – 11.80 | 90 | 12.97 | 10.29 – 15.65 | 122 | 17.58 | 14.46 – 20.70 |
| **Cumulative incidences of breast cancers stratified by age**  Age < 65 years: 522,722 in the unvaccinated group and 2,090,888 in the vaccinated group.  Age 65 – 74 years: 40,213 in the unvaccinated group and 160,852 in the vaccinated group.  Age ≥ 75 years: 32,072 in the unvaccinated group and 128,288 in the vaccinated group. | | | | | | | | | | | | | | | | |
| **V** | **Age** | **One month** | | | **Three months** | | | **Six months** | | | **Nine months** | | | **One year** | | |
|  |  | **Event** | **I** | **95% CI** | **Event** | **I** | **95% CI** | **Event** | **I** | **95% CI** | **Event** | **I** | **95% CI** | **Event** | **I** | **95% CI** |
| No | < 65 | 15 | 0.55 | 0.27 – 0.83 | 50 | 1.84 | 1.33 – 2.35 | 121 | 4.44 | 3.65 – 5.24 | 217 | 7.97 | 6.91 – 9.03 | 293 | 10.76 | 9.53 – 11.99 |
| Yes |  | 97 | 0.89 | 0.71 – 1.07 | 355 | 3.26 | 2.92 – 3.60 | 733 | 6.73 | 6.24 – 7.22 | 1110 | 10.19 | 9.59 – 10.79 | 1478 | 13.57 | 12.88 – 14.26 |
| No | 65 – 74 | 3 | 1.28 | 0.00 – 2.73 | 7 | 2.99 | 0.78 – 5.20 | 29 | 12.39 | 7.88 – 16.89 | 41 | 17.51 | 12.16 – 22.87 | 51 | 21.79 | 15.81 – 27.76 |
| Yes |  | 11 | 1.17 | 0.48 – 1.87 | 44 | 4.70 | 3.31 – 6.09 | 94 | 10.04 | 8.01 – 12.07 | 136 | 14.52 | 12.08 – 16.96 | 180 | 19.22 | 16.42 – 22.03 |
| No | ≥ 75 | 1 | 0.45 | 0.00 – 1.34 | 3 | 1.35 | 0.00 – 2.89 | 7 | 3.16 | 0.82 – 5.50 | 14 | 6.32 | 3.01 – 9.63 | 22 | 9.93 | 5.79 – 14.08 |
| Yes |  | 16 | 1.81 | 0.92 – 2.69 | 33 | 3.73 | 2.45 – 5.00 | 62 | 7.00 | 5.26 – 8.74 | 79 | 8.92 | 6.95 – 10.88 | 102 | 11.52 | 9.28 – 13.75 |

Cumulative incidence was presented by incidences per 10,000 individuals. I, cumulative incidence.

**11. Table S10:** The detailed cumulative incidence on prostate cancer

**Table S10** Cumulative incidences of prostate cancers in the matched cohort between vaccinated and unvaccinated individuals

| **Cumulative incidences of prostate cancers**  Number: 595,007 in the unvaccinated group and 2,380,028 in the vaccinated group) | | | | | | | | | | | | | | | | |
| --- | --- | --- | --- | --- | --- | --- | --- | --- | --- | --- | --- | --- | --- | --- | --- | --- |
| **V** | | **One month** | | | **Three months** | | | **Six months** | | | **Nine months** | | | **One year** | | |
|  |  | **Event** | **I** | **95% CI** | **Event** | **I** | **95% CI** | **Event** | **I** | **95% CI** | **Event** | **I** | **95% CI** | **Event** | **I** | **95% CI** |
| No | | 5 | 0.18 | 0.02 – 0.34 | 17 | 0.61 | 0.32 – 0.90 | 39 | 1.41 | 0.97 – 1.85 | 65 | 2.35 | 1.78 – 2.92 | 88 | 3.18 | 2.51 – 3.84 |
| Yes | | 43 | 0.39 | 0.27 – 0.50 | 137 | 1.24 | 1.03 – 1.44 | 288 | 2.60 | 2.30 – 2.90 | 438 | 3.95 | 3.58 – 4.32 | 599 | 5.40 | 4.97 – 5.84 |
| **Stratified by vaccine type**  cDNA vaccine: 333,698; mRNA vaccine: 1,928,363; Heterologous vaccination: 117,967 | | | | | | | | | | | | | | | | |
| cDNA vaccine | | 16 | 1.12 | 0.57 – 1.67 | 61 | 4.27 | 3.20 – 5.34 | 125 | 8.75 | 7.22 – 10.29 | 199 | 13.94 | 12.00 – 15.87 | 277 | 19.40 | 17.12 – 21.68 |
| mRNA vaccine | | 27 | 0.29 | 0.18 – 0.41 | 76 | 0.83 | 0.64 – 1.01 | 161 | 1.76 | 1.48 – 2.03 | 235 | 2.56 | 2.23 – 2.89 | 315 | 3.43 | 3.06 – 3.81 |
| Heterologous | | 0 | 0.00 | 0.00 – 0.00 | 0 | 0.00 | 0.00 – 0.00 | 2 | 0.41 | 0.00 – 0.98 | 4 | 0.82 | 0.02 – 1.63 | 7 | 1.44 | 0.37 – 2.51 |
| **Cumulative incidences of prostate cancers stratified by age**  Age < 65 years: 522,722 in the unvaccinated group and 2,090,888 in the vaccinated group.  Age 65 – 74 years: 40,213 in the unvaccinated group and 160,852 in the vaccinated group.  Age ≥ 75 years: 32,072 in the unvaccinated group and 128,288 in the vaccinated group. | | | | | | | | | | | | | | | | |
| **V** | **Age** | **One month** | | | **Three months** | | | **Six months** | | | **Nine months** | | | **One year** | | |
|  |  | **Event** | **I** | **95% CI** | **Event** | **I** | **95% CI** | **Event** | **I** | **95% CI** | **Event** | **I** | **95% CI** | **Event** | **I** | **95% CI** |
| No | < 65 | 2 | 0.08 | 0.00 – 0.19 | 4 | 0.16 | 0.00 – 0.32 | 6 | 0.24 | 0.05 – 0.43 | 14 | 0.56 | 0.27 – 0.85 | 19 | 0.76 | 0.42 – 1.10 |
| Yes |  | 8 | 0.08 | 0.02 – 0.14 | 24 | 0.24 | 0.14 – 0.34 | 66 | 0.66 | 0.50 – 0.82 | 101 | 1.01 | 0.81 – 1.20 | 144 | 1.44 | 1.20 – 1.67 |
| No | 65 – 74 | 3 | 1.79 | 0.00 – 3.81 | 6 | 3.57 | 0.71 – 6.43 | 17 | 10.12 | 5.31 – 14.92 | 25 | 14.88 | 9.05 – 20.71 | 36 | 21.42 | 14.43 – 28.42 |
| Yes |  | 14 | 2.08 | 0.99 – 3.17 | 51 | 7.59 | 5.51 – 9.67 | 98 | 14.58 | 11.70 – 17.47 | 158 | 23.51 | 19.85 – 27.17 | 215 | 31.99 | 27.72 – 36.26 |
| No | ≥ 75 | 0 | 0.00 | 0.00 – 0.00 | 7 | 7.05 | 1.83 – 12.27 | 16 | 16.12 | 8.23 – 24.01 | 26 | 26.19 | 16.14 – 36.24 | 33 | 33.24 | 21.92 – 44.56 |
| Yes |  | 21 | 5.29 | 3.03 – 7.55 | 62 | 15.61 | 11.73 – 19.50 | 124 | 31.22 | 25.74 – 36.71 | 179 | 45.07 | 38.49 – 51.66 | 240 | 60.44 | 52.81 – 68.06 |

Cumulative incidence was presented by incidences per 10,000 individuals. I, cumulative incidence.
